# Myeloid-Derived Suppressor Cells in Solid Tumors

**DOI:** 10.3390/cells11020310

**Published:** 2022-01-17

**Authors:** Tianmiao Ma, Bernhard W. Renz, Matthias Ilmer, Dominik Koch, Yuhui Yang, Jens Werner, Alexandr V. Bazhin

**Affiliations:** 1 Department of General, Visceral and Transplant Surgery, Ludwig-Maximilians-University Munich, 81377 Munich, Germany; Tianmiao.Ma@med.uni-muenchen.de (T.M.); Bernhard.renz@med.uni-muenchen.de (B.W.R.); Matthias.ilmer@med.uni-muenchen.de (M.I.); Dominik.koch@med.uni-muenchen.de (D.K.); Jens.werner@med.uni-muenchen.de (J.W.); 2 German Cancer Consortium (DKTK), Partner Site Munich, 81377 Munich, Germany; 3 Cancer Center, Union Hospital, Tongji Medical College, Huazhong University of Science and Technology, 1277 Jiefang Avenue, Wuhan 430022, China; yuhui.yang@yahoo.com; 4 Bavarian Cancer Research Center (BZKF), 91054 Erlangen, Germany

**Keywords:** myeloid-derived suppressor cells (MDSCs), solid tumor, immunotherapy, tumor microenvironment (TME)

## Abstract

Myeloid-derived suppressor cells (MDSCs) are one of the main suppressive cell population of the immune system. They play a pivotal role in the establishment of the tumor microenvironment (TME). In the context of cancers or other pathological conditions, MDSCs can differentiate, expand, and migrate in large quantities during circulation, inhibiting the cytotoxic functions of T cells and NK cells. This process is regulated by ROS, iNOS/NO, arginase-1, and multiple soluble cytokines. The definition of MDSCs and their phenotypes in humans are not as well represented as in other organisms such as mice, owing to the absence of the cognate molecule. However, a comprehensive understanding of the differences between different species and subsets will be beneficial for clarifying the immunosuppressive properties and potential clinical values of these cells during tumor progression. Recently, experimental evidence and clinical investigations have demonstrated that MDSCs have a close relationship with poor prognosis and drug resistance, which is considered to be a leading marker for practical applications and therapeutic methods. In this review, we summarize the remarkable position of MDSCs in solid tumors, explain their classifications in different models, and introduce new treatment approaches to target MDSCs to better understand the advancement of new approaches to cancer treatment.

## 1. Introduction

In recent decades, myeloid-derived suppressor cells (MDSCs) have been recognized as an indispensable cell population of the innate immune system, highlighted by their strong immunosuppressive activity in cancers and other pathological conditions, altering our adaptive immune response.

MDSCs are a heterogeneous group of immature myeloid cells derived from the bone marrow hematopoietic precursor cells [1]. In healthy individuals, immature myeloid cells (IMCs) differentiate into granulocytes, macrophages, and dendritic cells [2], and enter the corresponding organs and tissues, exerting normal immune functions. However, in pathological and chronic inflammatory conditions, such as cancers, infectious diseases, autoimmune disorders, or sepsis, a block in the normal myeloid differentiation occurs, and the persistent stimulation of myelopoiesis results in the expansion of MDSCs [3,4]. This is regulated by several factors, including, but not limited to, granulocyte/macrophage colony-stimulating factor (GM-CSF), interleukin (IL)-6, IL-10, IL-12, cycloxygenase-2 (COX-2), prostaglandin E2 (PGE2), and vascular endothelial growth factor (VEGF) [5,6]. When MDSCs accumulated and migrated to the periphery, their number and proportion increased by 10-fold, accounting for approximately 10% of peripheral blood mononuclear cells (PBMCs) [7] throughout the entire process of disease. These cells inhibit the normal immune function of T cells in the tumor microenvironment (TME) [8].

MDSCs show a wide range of phenotypes and are involved in the setting of tumor progression. In mice, MDSCs are marked by CD11b and Gr1, and can be divided into two subgroups, including polymorphonuclear MDSCs (PMN-MDSCs) with CD11b^+^ Ly6G^hi^ Ly6C^−/low^ and monocytic MDSCs (M-MDSCs) with CD11b^+^ Ly6G^−/low^ Ly6C^hi^ [9]. However, the cognition of MDSCs in humans is complicated and remains controversial. To imitate the same findings in mice, human MDSCs were specified by surface markers [10]. Human M-MDSCs are defined as CD14^+^ CD33^+^ CD11b^+^ HLA-DR^−^, and PMN-MDSCs as CD15^+^ CD33^+^ CD11b^+^ HLA-DR^−^ [11]. Both M-MDSCs and PMN-MDSCs can restrain T cells and NK cells by secreting reactive oxygen species (ROS), nitrogen oxide (NO), arginase-1, etc., and inducing regulatory T cells (Tregs) in some circumstances, therefore, suppressing the immune response of the host.

The special feature of MDSCs is their potent inhibitory function, which establishes an immunosuppressive TME that is conducive to tumor immune escape, and further promotes the occurrence and development of tumors. Hence, these cells are worth noting in future clinical immunotherapy applications.

## 2. MDSCs as the Main Component of Cellular Immune Suppressors

Tregs and MDSCs are considered two crucial immunosuppressive cell populations, participating in accelerating the progression and metastasis of tumors. Tregs contribute mainly to cellular immunity through direct contact between cells, secretion of inhibitory factors, and competition with antigen-presenting cells (APC) for costimulatory molecules [12]. Similarly, a broad range of suppressive indicators in MDSCs prevent immune cells’ (mostly T cells and NK cells) anti-tumor reactivity and support disease progression.

### Mechanisms of MDSCs Immunosuppressive Function

There are several mechanisms that consist of MDSC-mediated immunosuppression (Table 1). Firstly, MDSCs exhibit immunosuppressive effects under oxygen pressure, producing ROS and peroxynitrite (ONOO^−^). In the biological context, ROS take part in maintaining homeostasis [13], while in oxidative stress conditions, increased ROS concentration will hamper T-cell signaling activation, proliferation, and viability, thereby damaging the adaptive immune response [14,15]. ONOO^−^ is a strong nitrifying agent, ascribed to the reaction with superoxide and nitrogen oxides (NO) [16], and enriched in MDSCs and tumor cell aggregation sites [17,18]. It can nitrate T cell receptor (TCR) and CD8 molecules during direct contact between MDSCs and T cells and inhibit the proliferation of CD8^+^ T cells, causing the down-regulation of the immune activity [19,20]. MDSCs express nitrogen-oxygen synthase 2 (iNOS), which releases NO. The up-regulated expression of iNOS leading to NO production was proven to be relevant for obstructing IFN-γ signaling. This effect is due to a signal transducer and activator of transcription 1 (STAT1) in the anti-tumor immune response [21]. NO also directly impairs T cell function by inhibiting JAK/STAT5 pathways, MHC class II expression, and inducing T cell and NK cell apoptosis in tumor cells [22].

Secondly, MDSCs facilitate the consumption of some amino acids needed in our body. Both IL-4 and IL-13 can bind to IL-4Rα, inducing STAT6 activation and arginase-1 expression in myeloid cells (e.g., MDSCs and activated macrophages) [23]. The growing number of arginase-1 generated by MDSCs depletes L-arginine, leading to the G0–G1 phase being blocked during T cell proliferation and the low expression of the CD3ζ chain of the TCR, connected with tumor escape in vivo [24]. Human CD4^+^ T cells depend on the uptake of exogenous cystine/cysteine for glutathione and DNA synthesis [25,26]. MDSCs, however, prevent T cell activation via depriving cystine and restricting the availability of cysteine [27]. Indoleamine 2,3-dioxygenase (IDO) metabolizing tryptophan is a part of the malignant transformation process. The overexpression of IDO has been referred to support MDSCs in immunosuppressive TME influencing the inflammatory environment [28]. In clinical trials, IDO inhibitors have been used as a regimen for reducing MDSCs and abolishing their suppressive function on effector T cells [29].

Thirdly, MDSCs apply their immunosuppressive capability in tumor progression by multiple molecules. IL-10 produced by cancer and stroma cells hinders the maturation of myeloid cells by activating STAT3. These immature myeloid cells become MDSCs and impose immunosuppressive effects on CD8^+^ T cells [30]. In a renal cell carcinoma (RCC) a low level of IL-1β may influence adaptive and innate immune resistance on account of increasing MDSC infiltration [31]. IL-1β was also shown to provoke IL-10 production by MDSCs. In murine models, IL-6 was proven to facilitate MDSC expansion and activation, preventing the immune response directly [32]. GM-CSF allows the in vitro generation of MDSCs retaining their suppressive activity in vivo [33]. Tumor-derived G-CSF induced the overexpression of MDSCs, which are responsible for T cell suppression, tumor growth, and metastasis in gynaecological solid tumors [34,35]. It has been shown that transforming growth factor β (TGF-β) was contained in the regulation of hematopoiesis, which can interrupt the maturation of myeloid cells, prompting MDSC generation and expansion [36]. The production of TGF-β in CD11b^+^ Gr-1^+^ myeloid cells is one of the mechanisms of blocking cytotoxic T cell-mediated tumor immunosurveillance [37]. MDSC accumulation depends on CC chemokine receptor (CCR) 2-mediated signals. Bone marrow-derived CCR2^+^ MDSCs may affect the transport of T cells to the tumor site, supporting tumor growth [38]. MDSCs are also known to produce vascular endothelial growth factors (VEGF) that promote neo-angiogenesis and create a pre-metastatic environment, prolonging innate immunity suppression [39,40]. Interferon-γ (IFN-γ) and Toll-like receptor (TLR) 2 were reported to enhance the mediated immunosuppression of M-MDSCs, (not G-MDSCs), negatively manipulating the anti-tumor T cell response [41]. Prostaglandin E2 (PGE2) and cycloxygenase-2 (COX-2) are inflammatory mediators produced by different tumors [42]. PGE2 was confirmed to induce the accumulation of MDSCs, and inhibit the activation of CD4^+^ and CD8^+^ T cells [43]. Tumor-derived COX-2 conduces arginase-1 induction of MDSCs, resulting in tumor immune tolerance [44,45]. ADAM17 (a disintegrin and metalloproteinase domain 17), expressed on MDSCs, is likely to down-regulate L-selection on CD4^+^ and CD8^+^ T cells and prevent them to migrate to peripheral lymph nodes and tumor sites [5]. Furthermore, some small single-stranded non-coding RNA, such as microRNA-21 and microRNA-155, were also shown to adjust the immune suppression system mediated by MDSCs in tumors [46,47].

Lastly, cell-to-cell contact is another way for MDSCs to suppress the immune response. In the peripheral blood (PB) of pregnant women, M-MDSCs have been observed to significantly suppress T cell responses in a reactive oxygen species-dependent manner and require a mechanism that relies on cell contact [48]. Recently, one report demonstrated that MDSCs could paralyze T cells through the cell–cell transfer of the metabolite methylglyoxal, which may reduce T cell anti-tumor immunity [49]. The interplay between MDSCs and Tregs contribute to the establishment of an immunosuppressive environment. MDSCs promote the production of Tregs via a cell-contact dependent mechanism, inducing immune tolerance under conditions of cancers or other abnormal immune responses [50,51]. In addition, MDSCs also directly incite tumor-associated macrophages (TAMs) to facilitate immunosuppression [52]. Thus, the involvement of MDSCs is regarded as an obstacle to the immune response against solid tumors as well as hematological malignancies. Furthermore, the reciprocal connection between MDSCs and other immune cells is also of great value in tumor promotion.

**Table 1:** Main mechanisms of the MDSCs immunosuppressive function.

Mechanisms		Main Factors	Immune Response	References
Oxidative stress		ROS	Inhibit T cell activation, proliferation, and viability	[14,15]
		iNOS/NO	Impair T cell function and induce T cell and NK cell apoptosis	[19,20]
		ONOO-	Nitrate TCR/CD8 molecules and inhibit the proliferation of CD8+ T cells	[21,22]
Amino acid consumption	Arginine depletion	Arginase-1	Block G0-G1 phase during T cell proliferation	[23,24]
	Cystine deprivation	Cystine	Prevent T cell activation	[25,26,27]
	IDO overexpression	IDO	Influence the function of effector T cells	[28,29]
Typical Cytokines and other mediators support		IL-1β, IL-6, IL-10	Impose immunosuppressive effects on T cells	[30,31,32]
		GM-CSF	Suppress T cells	[33,34,35]
		TGF-β	Block cytotoxic T cell-mediated tumor immunosurveillance	[36,37]
		CCR2	Affect the transport of T cells to the tumor site	[38]
		VEGF	Prolong the innate immunity suppression	[39,40]
		IFN-γ	Negatively manipulating anti-tumor T cell response	[41]
		TLR2	Negatively manipulating anti-tumor T cell response	[41]
		PGE2 (COX2)	Inhibit the activation of CD4+ and CD8+ T cells	[43,44,45]
		ADAM17	Inhibit T cell migration to peripheral lymph nodes and tumor sites.	[5]
		miRNA-155, miRNA-21	Inhibit helper T cell and cytotoxic T cell proliferation	[46,47]
Cell–cell transfer		Other immune cells	Reduce T cell anti-tumor immunity	[48,49,50,51,52]

## 3. Crosstalk between MDSCs and Other Immune Cells

### 3.1. Conventional T Cells

Generally, MDSCs immunosuppressor function lies in their ability to restrain T cell proliferation and activation, as well as to impede T cell recruitment to lymph nodes or tumor tissues [19]. This process has been already introduced in the previous section. Conversely, inflammatory molecules, such as IFN-γ, which is released by activated T cells, could also trigger MDSC expansion, migration, and activation at the tumor site, resulting in the growth of solid tumors [53].

The production of IFN-γ and IL-13 by CD8^+^ T cells could be integrated into MDSCs, having a negative impact on the adaptive immune system [54]. Blocking IFN-γ and IL-13 in combination therapy of tumor–host could obtain the additive effect. Nagaraj et al. [55] reported that CD4^+^ T cells cause the conversion of MDSCs from antigen-specific to non-specific suppressors via retrograde MHC class II signaling. This characterization is important for understanding the nature of immune defects in cancer. Pinton et al. [56] identified that MDSCs only exhibit their immunosuppression ability on activated but not on resting T cells. This cell–cell interaction induces the release of IL-10 in T cells and in turn, activates STAT3 phosphorylation on MDSCs, enhancing the immunosuppressive activity of these cells. 

As critical members of the immune system, T cells are required for guiding the immune response process and controlling cancer development. The complex, in-depth mechanism of interaction between MDSCs and T cells presents a substantial challenge for therapeutic targeting.

### 3.2. Tregs and Th17

MDSCs and Tregs are both implicated in tumor escape from immunosurveillance. Immunosuppression can be weakened by decreasing the MDSCs and Treg cell populations in solid tumors after removing the tumor burden [57].

A few reports indicated that MDSCs are related to the induction of Treg [57,58] by direct contact. The high expression of IDO and arginase-1 in MDSCs fostered Treg expansion and differentiation [59,60]. In contrast, Tregs have also been shown to influence the survival and/or proliferation of MDSCs due to the production of certain cytokines. Lee et al. [61] demonstrated that Tregs control the regulation of MDSCs via TGF-β during murine colitis. Fujimura et al. [62] also signified that in melanoma that the depletion of Tregs lessened the production of IL-10 by MDSCs, readily decreasing the suppressive function of MDSCs. The interconnection between Tregs and MDSCs was dedicated to the acquisition of immunosuppressive effects, which may be utilized to achieve clinical benefit.

Th17 cells tend to promote immune reactions in many pathological conditions. A high level of arginase-1, expressed by MDSCs, enhances Th17 polarization [63]. Some studies suggested that the recruitment of MDSCs at tumor sites are often accompanied by aggregation of Th17 cells [64,65,66]. However, the potential connection between these two cell types demands further systematic exploration.

### 3.3. B Cells

Nowadays, many studies have pay attention to the interaction between MDSCs and B cells in the progression of tumor diseases. Unfortunately, the mechanisms on how MDSCs affect B cells and whether they could be applied to all circumstances remain unclear. Some studies supply new points of view. Wang et al. [67] implied that the upregulation of IL-7 and STAT5 influenced by MDSCs in lung cancer hindered the proliferation and differentiation of B cells. Moreover, M-MDSCs could weaken the B cell immune response by several suppressive mediators such as arginase-1, ROS, NO, and IDO [68,69]. MDSCs also directly act on B cells to decline IgM and IgG production (thereby reducing their levels in serum) through the V-domain Ig suppressor of T-cell activation (VISTA) from mice infected with an immunodeficiency-causing retrovirus [70].

B-regulatory cells (B-regs) are a newly identified immunosuppressive cell population [71], which are not yet well investigated. However, these cells are deemed significant contributors to neoplastic disorders [72]. Bodogai et al. [73] indicated that cancer-induced Bregs fully mobilized the immunomodulatory and tumor-promoting function of MDSCs, which are manifested by the increase of ROS and NO, inhibiting the CD4^+^ and CD8^+^ T cell proliferation. Lee-Chang et al. [74] uncovered that MDSCs represented a major immunosuppressive cell compartment in glioblastoma (GBM) patients and mice. They could deliver membrane-bound programmed death-ligand 1 (PD-L1) to B cells by microvesicles, resulting in the transformation of naïve B cells to Bregs and inhibiting regular immune responses.

### 3.4. Natural Killer (NK) Cells

MDSCs could advance tumor progression by affecting the anti-tumor activities of NK cells. The reduction of NK cells is relevant to the marked increase of MDSCs, not Tregs [75,76]. 

Liu et al. [77] verified that the cell–cell contact between MDSCs and NK cells in tumor-bearing mice could inhibit the IL-2–mediated activation of NK cells and perforin production, decreasing the ability of NK cells to attack tumor cells. MDSCs could also block IFN-γ secretion by NK cells, and NO produced by MDSCs further interferes with NK Fc Receptor to inhibit the cytotoxicity of NK cells [78]. 

Greene et al. [79] stated that the prevention of MDSC trafficking with a CXCR1/2 small molecule inhibitor can improve the therapeutic efficacy of adoptively transfer of NK cells, which clarified the important ability of MDSC to suppress NK cell function in TME. The PD-1/PD-L1 pathway has been validated to be the main index in cancer immunotherapy. A recent study reported a novel NK cell line, PD-L1, targeting high-affinity natural killer (t-haNK) cells, which retained its in vitro cytotoxic potency against various human cancer cells. These cells could effectively lyse tumor cells and MDSCs without affecting T and NK cells, relieving the immunosuppression of MDSCs in the TME [80]. Clinical trials should be implemented to ensure safety and efficacy in future applications.

### 3.5. Neutrophils

Neutrophils and G-MDSCs (or PMN-MDSCs) share the same origin and many morphological and phenotypic features [81]. Pillay et al. [82] proposed a hypothesis that G-MDSCs could be a unique phenotype of neutrophils. A few studies demonstrated that the tumor-promoting activity of neutrophils could be attributed to PMN-MDSCs. Semerad et al. [83] suggested that G-CSF was a principal regulator of neutrophil transport from bone marrow to blood, which was also confirmed to be involved in MDSCs suppression function. In addition, the production of ROS in both neutrophils and MDSCs is considered a major mechanism of immune suppression in the TME [84]. It appeared that G-MDSCs (or PMN-MDSCs) could represent neutrophils at distinct maturation stages, and the activated neutrophils are able to obtain the inhibitory activity of MDSCs.

### 3.6. Tumor-Associated Macrophages (TAMs)

In the case of chronic inflammation and tumors, the bidirectional nature of MDSC–macrophage interactions significantly amplified the level of IL-10 and declined the level of IL-6, IL-12 and MHC II, prompting the differentiation of CD4^+^ T cells (Th2), the absence of NK cells, and the development of Tregs [85]. 

Immature myeloid cells could rapidly differentiate into TAMs mediated by ROS elevation and STAT1 signaling activation [86,87]. Corzo et al. [88] made use of a tumor mouse model to explore the nature of tumor-associated MDSCs under hypoxia. Their data indicated that the existence of hypoxia-inducible factor (HIF) 1α directly controls the differentiation of MDSCs to TAMs at tumor sites. Kumar et al. [89] observed that the fate of M-MDSCs was modulated by the activation of STAT3. When this transcriptional factor was selectively inhibited by hypoxia-induced CD45 tyrosine phosphatase upregulation, M-MDSCs would differentiate into TAMs, which introduced a new mechanism for the design of targeted therapy. Loeuillard et al. [90] characterized that the blockade of TAMs did not reduce the tumor growth in cholangiocarcinoma (CCA) but supplied a compensatory accumulation of G-MDSCs. This MDSC subset could facilitate tumor invasion and metastasis. After the combined removal of TAM and G-MDSC populations, the effect of the immune checkpoint blockade (ICB) with anti–PD-1 was improved, supporting a prospective therapeutic approach on targeting both macrophages and MDSCs.

### 3.7. Dendritic Cells (DCs)

The cancer cell-induced immunosuppressive microenvironment does not only limit the activity of mature and functional DCs but also expand the tumor-promoting immature DC populations [91]. The recruitment of MDSCs seems to be linked to this impaired differentiation of DCs; however, the basic mechanisms are still not fully understood.

An early study evidenced that the overexpression of Ca^2+^ binding myeloid-related proteins (S100A8 and S100A9) induced by STAT3 could be one of the reasons for DC differentiation disorder and MDSC hyperproduction, enhancing the immunosuppression of a tumor-bearing host [92]. This underlying connection between immune cells and pro-inflammatory factors might interpret a pathway to negatively regulate the immune response in cancer. Another finding in melanoma patients showed that MDSCs did not affect the viability of DCs, but impaired DC maturity by restricting antigen uptake due to the blockage of DC capacity to stimulate IFN-γ-producing T cells, as well skewing DC cytokine production towards an anti-inflammatory phenotype [93]. Moreover, the highly suppressive M-MDSCs (CD14^+^ HLA-DR^−^ cells) were found to impair the quality of the overall DC vaccine; therefore, their removal could be beneficial [93]. The mutual transformation and interaction between MDSCs and DCs could be a focus of future immunotherapy research.

**Figure 1:**
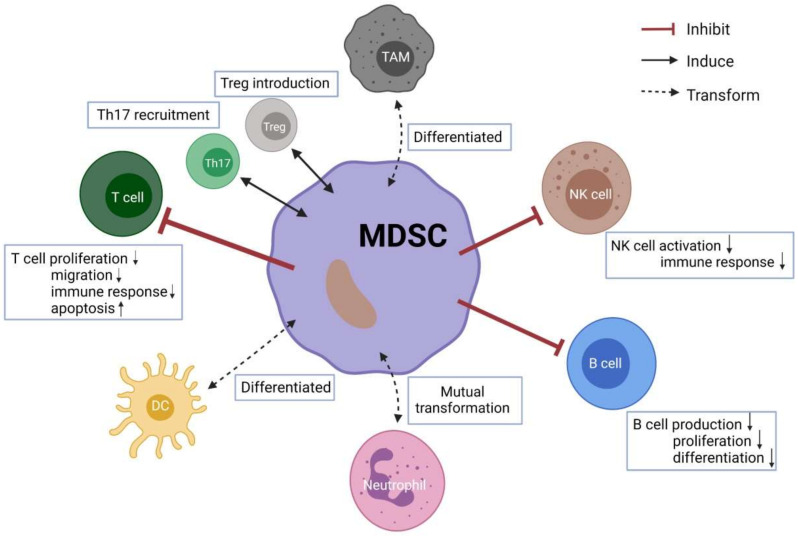
Crosstalk between MDSCs and other immune cells. Up arrows mean increased, and the down arrows mean decreased.

To summarize, given the ability of MDSCs to modulate various immune cells in facilitating tumor progression, this immune cell population may be an important therapeutic target. (Figure 1, created with BioRender.com (accessed on 29 November 2021)) Next, we will focus on their potential clinical application in both mouse models and human solid tumors.

## 4. Defining MDSCs in Mouse and Human

As mentioned previously, MDSCs are a very heterogenic group not only on the basis of the cellular origin but also in the context of their surface markers. In mice, MDSCs present high levels of CD11b (a subunit of the β2 integrin Mac-1) and Gr1 (granulocytic marker) [94]. Gr1 antigen detected by the RB6-8C5 antibody was discovered to be made up of two phosphatidylinositol-anchored cell surface glycoproteins, Ly6C and Ly6G [95]. Based on their expression, murine MDSCs can be classified into two subtypes, monocytic MDSCs (M-MDSCs) and polymorphonuclear MDSCs (PMN-MDSCs), also known as granulocytic MDSCs (G-MDSCs). M-MDSCs display a high levels of Ly6C expression while low or no expression of Ly6G, resembling monocytes in phenotypical and morphological features, while PMN-MDSCs express low levels of Ly6C and high levels of Ly6G, similar to neutrophils [96]. Although these two subpopulations are both involved in immune suppression, current views assume that their proportions and inhibitory pathways in different tumors are not completely the same. M-MDSCs mainly up-regulate NO and arginase, secreting soluble cytokines [81], whereas G-MDSCs need to interact with T cells directly [97] and usually exert their suppressive function utilizing ROS. The nature of chemokines produced by tumor cells might support the infiltration of M-MDSCs in early tumor sites and transmission from the primary site [81], while G-MDSCs could facilitate tumor cell expansion and metastatic growth via reverting epithelial-mesenchymal transition (EMT)/cancer stem cell (CSC) phenotype [98]. A recent study published in 2021 provided evidence that endoplasmic reticulum (ER) stress response appears to be a dominant factor in surveillance the immune-suppressive activity of PMN-MDSCs by the IRE1α and ATF6 pathways in tumor-bearing mice. In contrast, the induction of M-MDSC depends on IFN-γR signaling instead of the ER stress response [99]. 

Unlike mouse MDSCs, human MDSCs are less well characterized due to the lack of Gr1; therefore, they are usually express high levels of CD33, CD11b surface markers and low levels of HlA-DR [100]. Similar to murine MDSCs, human MDSCs can also be subdivided into CD14^+^ M-MDSC and CD15^+^ PMN-MDSC populations [101,102]. Notably, another type of MDSCs with CD11b^+^ CD14^−^ CD15^−^ was defined as early-MDSCs (E-MDSCs) [9], comprising more immature progenitors.

At the moment, a debate on the subsets, origin, and functions of human MDSCs still exists; thus, other methods need to be discussed to clarify the characterization of human MDSCs. CCAAT/enhancer-binding protein (c/EBPβ) plays a key role in the generation of in vivo tumor-induced MDSCs [103]. Moreover, STAT3 signaling was revealed to promote MDSC differentiation and expansion [104]. Although these proteins are not surface markers and are therefore difficult for analysis, they helped to define two separate MDSC groups, CD33^+^ HLA-DR^−/low^ STAT3^+^ and CD11b^+^ HLA-DR^−/low^ c/EBPβ^+^, which provided novel diagnostic and therapeutic tools for cancer immunotherapy [105]. Other myeloid markers, such as PD-L1, CD40, CD49d, CD80, CD115, and CD124, were also discovered to describe specific patterns of MDSCs [106,107,108].

Since MDSCs are a group of continuums in different phases of differentiation, characterizing MDSCs subtypes in various model systems and species, especially distinguishing between G-MDSCs and neutrophil, M-MDSCs and TAMs or monocyte-derived dendritic cells (mDCs), would help us to comprehend their functions for distinct organisms and activation procedures under pathological conditions.

## 5. MDSCs in Murine Models of Solid Tumors

MDSCs are abundant in the TME and could be employed in disease prediction, clinical outcome evaluation, prognosis value analysis, and immunotherapies testing. Compared with human models, most early mechanistic and functional studies on MDSCs were carried out in mice. It has been reported that MDSCs can be detected from peripheral blood [109], lymph nodes [110], spleen [111], and tumor sites [112] in murine tumor models.

### 5.1. Functional Studies in Mice

In various tumor-bearing mouse models, MDSCs increased with tumor progression, also in some premalignant states, but were less infiltrated in normal tissues [50,113,114]. Karakhanova et al. [115] demonstrated a pronounced accumulation of MDSCs in tumor and spleen of the tumor-bearing mice in an orthotopic mouse model of pancreatic ductal adenocarcinoma (PDAC). These cells were highly immunosuppressive and were associated with VEGF enrichment during PDAC progression. Meyer et al, using a melanoma mouse model, have shown that accumulated MDSCs inhibited tumor-reactive T cells and down-regulated their TCR ζ-chain [116]. Siret et al. [50] analyzed MDSC populations in PDAC mice by immunohistochemistry (IHC) and multiparametric flow cytometry. It was observed that the expression of B7-H1 (PD-L1) and CD40 (markers involved in MDSC-mediated immunosuppression) were up-regulated on the two MDSC subgroups both in tumor and in the spleen. However, F4/80 and CD124 (MDSCs associated surface markers) displayed increased level in M-MDSCs than G-MDSCs. Additionally, the quantity of ROS and arginase-1 were more ascendant in tumor mice than the naïve controls, showing a strong suppressive capacity against CD8+ T cells. This indicated that it is necessary to identify MDSCs and their detailed classification in cancer, and the recruitment and activation of these cells is a hallmark of abnormal immune function.

Metastasis is one of the leading causes of death in patients with solid cancer. PMN-MDSCs preferentially accumulated in melanoma mice, and then disseminated cancer cells to lymph nodes and lung by inducing EMT of tumor cells [117], establishing a favorable microenvironment for tumor migration. Hamilton et al. [118] found in mice bearing metastatic 4T1 or 4TO7 murine mammary tumors, that the pulmonary accumulation of MDSCs could promote metastatic tumor growth. Bosiljcic et al. [119] also examined the high level of MDSCs in the lungs, metastases from primary 4T1 tumors, and confirmed that the surgical removal of primary tumor diminished the quantity of G-MDSCs and M-MDSCs, as well as the metastatic growth in the lungs.

Stress response after surgical trauma has a link to a poor clinical outcome. The amplification of iNOS/NO released by MDSCs was sensitive in injury-induced immune dysfunction, and using a potent NO scavenger could protect against lymphocyte changes following mouse abdominal surgery trauma [120]. Highly suppressive MDSCs expanded after operation in the melanoma mouse model, and impaired the proliferation and functions of T cells, resulting in postoperative cancer recurrence [121].

Taken together, MDSC expansion represents immunological deficits and negative effects in mouse tumor models, and has shown potential use as a metastatic marker. However, more in-depth investigations should be conducted to explore the value of the targeting MDSCs in clinical application.

### 5.2. Therapy Testing in Mice

Several studies have mentioned that limiting the numbers of MDSCs and/or their immunosuppressive ability could restore the function of T cells and delay tumor progression. To target MDSCs, we can take measures from the following aspects: (i) promote the differentiation of MDSCs; (ii) block the expansion and accumulation of MDSCs; (iii) inhibit the immunosuppressive function of MDSCs; and (iv) reduce the numbers of MDSCs (Table 2).

The differentiation of MDSCs into DCs or macrophages is achievable in many experiments. In vivo, Very Small Size Proteoliposomes (VSSP) were verified to differentiate MDSCs into phenotypically mature DCs, diminishing MDSCs immunosuppression effects in tumor-bearing mice [122]. All-trans retinoic acid (ATRA), a derivative of vitamin A, has a strong ability in inducing the differentiation of MDSCs into DCs and/or macrophages in vitro, primarily via the neutralization of high ROS production and up-regulating the protein level of glutathione synthase (GSS) in these cells [123]. This has also helped to improve the efficacy of other therapies, such as chimeric antigen receptor (CAR) therapies [124], antiangiogenic therapies (ATT) [125], DC vaccinations [126], and so on. Varikuti et al. [127] found that ibrutinib can reprogram MDSCs to mature DCs using an orthotopic mouse breast cancer model, which at least in part improved their anti-tumor immunity. A systematic review concluded some preclinical studies and publications to assess ibrutinib’s efficacy in cell lines and animal models of gynecological malignancies [128]. Their results also prove that this drug could regulate the cytokine production, PD-L1 expression, as well as MDSC and other myeloid cell movement, which could be a promising therapy on solid tumors. Additionally, chloroquine (CQ) [129] and docetaxel [130] both strengthen the anti-tumor immune response by resetting MDSCs toward a M1 macrophages-like phenotype, exerting pro-inflammatory and phagocytic functions.

The expansion and accumulation of MDSCs rely on tumor-derived factors; thus accordingly, the depletion of these elements would be beneficial for increasing the effectiveness of clinical therapies on MDSCs. Melani et al. [131] showed in breast cancer mice that amino-biphosphonates could stop MDSCs expansion by inhibiting MMP-9 activity and overcoming tumor-induced immune suppression. Sun and her colleagues [132] proposed that SX-682, a small-molecule inhibitor of CXCR1 and CXCR2, obstructs MDSCs trafficking while enhancing T cell immunotherapy in syngeneic models of oral and Lewis lung carcinoma. VEGF receptor tyrosine kinase inhibitors (TKI) have been implicated in dealing with solid tumors, but drug resistance is still a clinical management problem [133]. Increased MDSCs in TME was believed to be correlated with resistance to immune therapies [134,135]. Diaz-Montero et al. [136] investigated treatment with sunitinib (a member of VEGF TKI class) and a MEK inhibitor in renal cell carcinoma (RCC) patient-derived xenograft (PDX) mouse models, concluding that decreasing the level of G-CSF and the accumulation of G-MDSCs, significantly delaying drug resistance. PD-1 is highly expressed in myeloid cells induced by lipopolysaccharide (LPS) via the NF-κB signaling pathway [137]. Strauss et al. [138] revealed that the myeloid- but not T cell-specific deletion of PD-1 in different mouse models prevented the accumulation of granulocyte/macrophage progenitors (GMPs) and MDSCs, whereas the systemic output of myeloid effector cells and T effector memory cells (Tem) with the improved anti-tumor function was increased. This study pointed out that myeloid-specific PD-1 blockade mediates myeloid cell-intrinsic effects, promoting a systemic anti-tumor response. 

Another strategy aims to block MDSCs immunosuppressive functions and revoke the immune activity of T cells. For example, COX-2 inhibitors, such as celecoxib, could minimize the production of PGE2 and ROS in solid tumor development, diminishing the suppression ability of MDSC subpopulations and reinforcing the infiltration of CD8^+^ T cells [139,140]. Yokoi et al. [141] also used a mouse model of endometrial cancer to demonstrate that celecoxib could inhibit the MDSC activity by cancelling the increase of IL-6 production and restraining tumor progression. Phosphodiesterase-5 (PDE-5) inhibitors, such as sildenafil or tadalafil, have been reported to reduce the expression of arginase-1 and iNOS and thus inhibit the immune escape mechanism mediated by MDSCs while restoring T cell proliferation and inducing tumor cell apoptosis [142,143,144]. Likewise, nitro-aspirin blunted both iNOS and arginase activity in tumor-associated MDSCs and corrected immune dysfunction in colon cancer mouse models [145].

In addition, some clinically approved drugs have been applied to deplete MDSCs. Suzuki et al. [146] explored the potential use of gemcitabine in anti-tumor immune activity, indicating that this chemotherapeutic agent could eliminate splenic MDSCs in tumor-bearing mice. Recently, gemcitabine has been identified as an MDSC depleting chemotherapeutic agent in various cancers. The drug can reduce the expression of immunosuppressive mediators, including TGF-β, IL-6, and IL-10, promoting the consumption of MDSCs [147]. At the same time, several studies have reported that the combination of gemcitabine and capecitabine (a 5-FU pro-drug) could more effectively lower the MDSC levels and exert higher anti-tumor effects, which may also enhance the efficacy of other treatments [148,149,150]. Aboalsoud et al. [151] used a low dose of naltrexone (LDN) in solid Ehrlich carcinoma in mice to investigate its impact on tumor growth. The results implied that LDN could reduce MDSC count and tumor weight, suggesting that LDN could be potentially beneficial in clinical practice. Synthetic Nanoparticle Antibodies (SNAbs) were developed to efficiently deplete MDSCs. The systemic injection of MDSC-targeting SNAbs in a mouse triple-negative breast cancer model was affirmed to selectively lower the circulating MDSCs and promote T cell and NK cell infiltration into the tumor [152].

**Table 2:** MDSCs-mediated immunotherapy strategies in solid tumors.

Treatment Strategies	Representative Treatment	Cancer	Factors	References
Promote the differentiation of MDSCs	Very Small Size Proteoliposomes (VSSP)	Sarcoma	Arginase-1, Nos2	[122]
	All-trans retinoic acid (ATRA)	Sarcomas/Breast cancer/Pancreatic cancer	ERK pathway, ROS	[123,124,125,126]
	Ibrutinib	Breast cancer	VEGF, MMP9, CXCL1	[127,128]
	Chloroquine	Melanoma/Hepatocarcinoma	Arginase-1, iNOS, IDO-1, IL-10, TGF-β	[129]
	Docetaxel	Breast cancer	ROS, IL-10, IL-12	[130]
Block the expansion and accumulation of MDSCs	Amino-biphosphonates	Breast cancer	VEGF, MMP9	[131]
	SX-682	Oral and Lewis lung carcinoma	CXCR1/2	[132]
	Sunitinib	Renal cell carcinoma	VEGF, G-CSF	[136]
	PD-1 ablation	Fibrosarcoma/Colon carcinoma/Melanoma	PI3K/Akt pathway, G-CSF	[137,138]
Inhibit the immunosuppressive function of MDSCs	Celecoxib	Glioma/Mesothelioma/Endometrial cancer	COX-2, PGE2, IL-6, G-CSF, ROS	[139,140,141]
	Sildenafil or Tadalafil	Breast cancer/Melanoma/Hepatocellular carcinoma	PDE5, arginase-1 and iNOS	[142,143,144]
	Nitro-aspirin	Colon cancer/Breast cancer	Arginase and iNOS	[145]
Reduce the numbers of MDSCs	Gemcitabine and 5-Fu	Melanoma/Lewis lung carcinoma/Colon cancer/Pancreatic cancer	TGF-β, IL-6 and IL-10, IL-1β	[146,147,148,150]
	Naltrexone	Solid Ehrlich carcinoma	IFN-γ	[151]
	Synthetic Nanoparticle Antibodies (SNAbs)	Breast cancer	S100A8/A9	[152]

Currently, therapeutic plans based on reversing immunosuppression of MDSCs, directly or indirectly, have increasingly achieved gratifying results. However, men are not similar to mice, and more studies are required to prove clinical safety and efficacy in future applications.

## 6. MDSCs in Patients with Solid Tumors

### 6.1. Clinical Importance of MDSCs in Humans

Human MDSCs and their phenotypes are not as well defined as in mice, but the clinical importance of these cells has been widely studied.

The accumulation of MDSCs is most pronounced in patients with stage IV, but can also be detected as early as in stage I patients [153]. Sieminska [113] reviewed that MDSCs can be detected throughout the course of colorectal cancer, especially related to tumor metastasis and poor prognosis. The same is true for melanoma [154,155], hepatocellular carcinoma (HCC) [156,157], and non-small cell lung carcinoma (NSCLC) [158,159] patients. Therefore, the idea occurs to use MDSCs as prognostic biomarkers. Concerning the prognostic value of MDSCs, some meta-analyses have already been published, which illustrated that patients with a large number of MDSCs tend to have poorer clinical outcomes [160,161,162,163]. One study [164] conveyed that elevated MDSCs in gastrointestinal cancers could serve as an independent prognostic factor for decreased survival, normally linked with an elevation of IL-13, arginase-1, and Tregs. Similar findings are also described in gynecological cancers [165,166]. Shimura et al. [167] indicated that immune suppression mediated by G-CSF-induced MDSCs could be the reason leading to the poor prognosis in gynecological cancer patients. Moreover, MDSC-induced immune suppression could facilitate metastasis in the circulation. The rising percentage and absolute number of MDSCs have an association with an increased metastatic tumor burden in patients with breast cancer [168] and colorectal cancer [169].

It is also essential to assess the different roles of two MDSC subtypes (M-MDSCs and G-MDSCs) in solid tumors. Circulating M-MDSCs increased in various cancers, associated with promoting tumor growth, and had a detrimental impact on the survival of patients with malignancies [154,156,170]. Yuan et al. [171] found that M-MDSCs can attenuate T cell proliferation and IFN-γ production and showed a certain correlation with clinical cancer staging and pathological grades. Wu et al. [172] characterized that M-MDSCs are the typical MDSCs in the PB and ascites from ovarian cancer (OC) patients. This phenomenon may be attributed to the overexpression of IL-6 and IL-10 and their downstream STAT3 signal. The higher level of M-MDSCs in OC patients indicated shorter recurrence-free survival. G-MDSCs are usually elevated in terminal cancer patients [173,174,175], accompanied by poor physical status and prognosis. In light of the differences between these two subgroups of MDSCs, it is necessary to consider their respective characteristics at different stages of the disease, which would be meaningful for predicting the development of cancer and evaluating the response of cancer patients to immunotherapy. Of particular note, the survival kinetics of M-MDSCs and G-MDSCs from human PBMCs after blood draw are not equal in terms of time point analysis. There is no obvious difference in G-MDSC levels over time, but M-MDSCs should be studied within 4 h after blood sampling [176]. Therefore, for analyzing the subsets of MDSCs in PBMCs, caution in the selection of appropriate time points is required.

Furthermore, MDSCs are incorporated in estimating therapeutic response in cancer patients, and the blockade of MDSCs would intensify the response to antiangiogenic therapy (AAT), chemotherapy, radiotherapy, or perioperative therapy [177,178].

### 6.2. Targeting of MDSCs for Clinical Applications

The clinical significance of MDSCs in cancer is established; therefore, approaches for selectively targeting MDSCs and their inhibitory activities to treat patients with solid tumors are under development. Many clinical trials on MDSCs are ongoing (Supplementary Tables S1 and S2).

Monoclonal antibodies (mAbs) against checkpoint inhibitors aiming to boost anti-tumor immunity have shown a great challenge in the combat against cancer and could improve outcomes even more in combination with other therapies. Pembrolizumab, a PD-1 blocking mAb, was approved to treat unresectable or metastatic solid tumors [179], such as head and neck squamous cell carcinoma (HNSCC) [180], NSCLC [181], and melanoma [182]. A phase 2 trial on the combination of pembrolizumab and BL-8040 (a CXCR4 antagonist) in metastatic PDAC showed that BL-8040 reduced MDSC numbers and increased effector T cell tumor infiltration, suggesting that BL-8040 could expand the benefit of pembrolizumab for PDAC patients [183]. Ipilimumab is a fully humanized mAb that potentiates the anti-tumor T-cell response by blocking cytotoxic T-lymphocyte-associated antigen 4 (CTLA-4) [184]. Sade-Feldman et al. [185] discovered that treating melanoma patients with anti-CTLA4 (ipilimumab) therapy could lower the frequency of MDSCs, which may change the suppressive environment and obtain better disease outcomes. Tobin et al. [186] combined ipilimumab with ATRA to target MDSCs in melanoma patients. Compared to ipilimumab treatment alone, adding ATRA to a standard of care ipilimumab appears to be safe, and ATRA may upgrade ipilimumab efficacy by reducing the frequency of MDSCs, increasing the activation of CD8^+^ T cells, as well as ameliorating the incidence of grade 3 or 4 adverse events. At the same time, a follow-up clinical trial is being recruited to appraise their potential safety and efficacy. 

Moreover, other mAbs, such as bevacizumab (NCT02669173), nivolumab (NCT03486119, NCT02917772), and atezolizumab (NCT03201458, NCT02992912), have also been extensively investigated to be added to clinical trials aiming at MDSC function, looking forward to introducing better treatments for patients with solid tumors.

At present, altering the production and biological functions of Tregs and MDSCs to decrease immunosuppression and improve solid tumor treatment has been adopted in preclinical and clinical trials. Gemcitabine can decrease G-MDSCs, but not M-MDSCs, and increase the ratio of effector T cells/Tregs, boosting the immune status of PDAC patients [187]. Tivozanib inhibits Tregs and MDSCs function by mediating c-Kit/SCF signaling, which reversed the tumor immune suppression and correlated with survival of hepatocellular carcinoma (HCC) patients [188]. Weed et al. [189] carried out a randomized phase 2 trial to evaluate the presence of MDSCs and Tregs in HNSCC patients before and after being treated with tadalafil. It seems that daily tadalafil treatment could reduce the infiltration of MDSCs and Tregs at the tumor site, promoting CD8^+^ T cell proliferation and activation and changing the tumor macro- and microenvironment. Another phase 2 trial in advanced urothelial carcinoma certified that cabozantinib, a multikinase inhibitor, could also be a new therapeutic option for these cancer patients by lowering the percentage of Tregs among CD4^+^ T cells and the G-MDSC populations [190].

Tumor-induced PD-L1 expression was restricted to the myeloid cells and, specifically, to the TAMs and MDSCs. One study suggested that CCA patients with a high level of TAMs or MDSCs seemed to be beneficial from immune checkpoint inhibitors (ICIs) [90]. Prima et al. [191] showed that in murine bladder carcinoma, PGE2-forming enzymes microsomal PGE 2 synthase 1 (mPGES1) and COX2 were involved in the regulation of PD-L1 expression on TAMs and MDSCs. Utilizing celecoxib targeting PGE 2 metabolism will help to diminish PD-L1 mediated immune suppression. Okla [192] demonstrated that PD-L1 expression on TAMs and M-MDSCs were increased in the blood of OC patients. However, no results can prove that PD-L1 is related to the prognosis and clinicopathologic parameters. These data should be concerned in future clinical studies/trials.

The co-dependency of MDSCs and DCs in tumor growth appears to be a potential member in immunotherapy. Kong et al. [193] showed that GM-CSF accelerated DCs and MDSCs response speed to pro-inflammatory stimuli differentially regulated via TLR4. These data unveil the substantial role of GM-CSF in a substantial initiation of immunity, with promising implications for future immunotherapy development based on DCs. A few clinical trials on DC vaccination are in progress (NCT01622933, NCT01808820, NCT02479230).

## 7. Conclusions

MDSCs accumulate, expand, and activate in cancer growth, playing a critical role in tumor maintenance and progression because of their powerful immunosuppressive functions. According to the preceding observations, it is reasonable to consider MDSCs as principal predictors of tumor prognosis and metastasis and to include them in future clinical therapeutic strategies. Not only can they provide more effective individualized treatment for patients with solid tumors, but they also amplify the efficiency of other therapies. Importantly, human MDSCs are more complex than mouse MDSCs; thus more attention should be paid to the possible effectiveness and safety of new drugs targeting MDSCs in clinical use. To address the limitations of current research, further studies should be conducted to fully understand the existence, heterogeneity, phenotypes, and adaptative conditions of MDSCs in practical application, bringing a more favorable prognosis for patients with malignant tumors.

## Data Availability

Not applicable.

## Supplementary Materials

The following supporting information can be downloaded at: https://www.mdpi.com/article/10.3390/cells11020310/s1, Table S1: List of clinical trials with published results targeting on MDSCs in solid tumors; Table S2: List of completed or recruiting clinical trials without results submitted targeting on MDSCs in solid tumors.
