# Supplementary material for: Myeloid-Derived Suppressor Cells in Solid Tumors
Source: Cells. 2022 Jan 17;11(2):310. doi: 10.3390/cells11020310 (PMC8774531; doi:10.3390/cells11020310)
Supplement: Supplementary file 1 [file cells-11-00310-s001.zip › cells-1510446-supplementary.pdf]

Supplementary Table S1. List of clinical trials with published results targeting on MDSCs in solid tumors

Author	PMID	Year	Tumor Type	No. of Patients	Treatments	Stage	Main results
Bruno.B	32451495	2020	Pancreatic cancer	59 patients	BL-8040 in combination with pembrolizumab and chemotherapy	COMBAT trial	BL-8040 decreased MDSCs in PDAC
Andrea.B.A	32645282	2020	Urothelial cancer	68 patients	Cabozantinib	phase 2 trial	Cabozantinib lowered MDSCs in advanced urothelial carcinoma
Yaewon.Y	26787822	2016	Solid tumors	27 patients	Ex vivo-expanded and highly activated NK cells (MG4101)	phase 1 trial	MG4101 reduced MDSCs in solid tumors
Tian.Z	32757302	2020	Urothelial cancer	75 patients	Pembrolizumab and acalabrutinib	phase 2 trial	BTK inhibition suppressed MDSCs and improved T-cell activation in metastatic urothelial cancer.
Andres.F	28050790	2017	Solid tumors	32 patients	DS-8273a	phase 1 trial	DS-8273a decreased MDSC in patients with advanced solid tumors
Haris.Z	31231785	2019	Bladder cancer	19 patients	Sunitinib	phase 2 trial	Sunitinib reversed MDSCs-mediated immunosuppression in patients with bladder cancer
Howard.L.K	19915919	2010	Melanoma	50 patients	Oncolytic herpesvirus encoding GM-CSF (Oncovex(GM-CSF))	phase 2 trial	GM-CSF downregulated MDSCs in patients with metastatic melanoma
Steven.E.F	20842055	2010	Kidney Cancer and Melanoma	21 kidney cancers 19 melanoma patients	High-dose intravenous interleukin-2 (IL-2) therapy	-	Patients with high levels of MDSCs either before starting IL-2 therapy or after therapy had less favorable outcomes
A.J.Montero	22138748	2012	Breast cancer	39 patients with 40 evaluable tumors	NOV-002 in combination with doxorubicin and cyclophosphamide followed by docetaxel	phase 2 trial	Patients with high MDSC levels could benefit from NOV-002 and standard chemotherapy in breast cancer
Lisa.H.B	31014399	2019	Melanoma	30 patients	Antigen-engineered DC vaccine	Phase 1 trial	Multiple antigen-engineered DC vaccines changed the frequency of MDSCs in patients with melanoma
Richard.P.T	30121453	2018	Advanced melanoma	10 patients	Ipilimumab monotherapy or Ipilimumab plus all-trans retinoic acid (ATRA)	phase 2 trial	ATRA decreased the frequency of MDSCs, activated CD8+ T cells and matured HLA-DR(+) myeloid cells.
David.M.P	31600167	2019	Glioblastoma	11 patients	Capecitabine + bevacizumab	Phase 0/I trial	Capecitabine+bevacizumab reduced circulating MDSC levels
Donald.T.W	25320361	2015	Head and neck squamous cell carcinoma	35 patients	Tadalafil	phase 2 trial	Tadalafil lowered MDSCs in patients with HNSCC

Supplementary Table S2. List of completed or recruiting clinical trials without results submitted targeting on MDSCs in solid tumors

Clinical trail number	Solid Tumor Type	No. of Patients	Treatment	Stage	Status
NCT03525925	Metastatic Solid Tumors	15 patients	Ibrutinib and Nivolumab	Phase 1	Active, not recruiting
NCT03673787	Glioblastoma, Multiforme Prostate Cancer	87 patients	Ipatasertib in Combination With Atezolizumab	Phase 1/2	Recruiting
NCT02637531	Advanced Solid Tumors	219 patients	IPI-549	Phase 1	Active, not recruiting
NCT02432963	Adult Solid Neoplasm	19 patients	P53MVA and Pembrolizumab	Phase 1	Active, not recruiting
NCT04105335	Advanced Solid Tumours	108 patients	MTL-CEBPA in Combination With a PD-1 Inhibitor	Phase 1	Recruiting
NCT04001101	MSI-H Solid Tumors	140 patients	RT and Anti-PD-1	Phase 2	Recruiting
NCT02922764	Advanced Solid Malignancies an d Lymphoma	135 patients	RGX-104	Phase 1	Recruiting
NCT02453620	Solid tumors	45 patients	Entinostat, Nivolumab, and Ipilimumab	Phase 1	Active, not recruiting
NCT02916745	Lung Cancer	5 patients	Photodynamic therapy-Photofrin	Phase 1	Active, not recruiting
NCT02526017	Advanced Solid Tumors	295 patients	Cabiralizumab in Combination With Nivolumab	Phase 1	Completed
NCT03961698	Triple-Negative Breast Cancer or Renal Cell Carcinoma	90 patients	IPI-549 Combined With Front-line Treatments	Phase 2	Recruiting
NCT04815720	Squamous Cell Carcinoma of the Head and Neck	80 patients	Pepinemab in Combination With Pembrolizumab	Phase 1/2	Recruiting
NCT04757662	Astrocytoma	16 patients	Tadalafil	Phase 2	Recruiting
NCT03161431	Metastatic Melanoma	77 patients	SX-682 in Combination With Pembrolizumab	Phase 1	Recruiting
NCT04129255	Neuroendocrine Tumors	34 patients	Octreotide LAR	Phase 2	Completed
NCT03486119	Non-Small Cell Lung Cancer	60 patients	Nivolumab	Phase 1/2	Completed
NCT02544880	Head and Neck Squamous Cell Carcinoma	16 patients	Tadalafil and MUC1 Vaccine	Phase 1	Completed
NCT03300817	Lung cancer	50 patients	MUC1 Vaccine	Phase 1	Recruiting
NCT02992912	Metastatic Tumours	187 patients	Atezolizumab With Stereotactic Ablative Radiotherapy	Phase 2	Recruiting
NCT04352777	Advanced ER+/HER2- Breast Cancer	30 patients	Endocrine Therapy and Abemaciclib	Phase 2	Recruiting
NCT01803152	Children and Adults With Sarcoma	19 patients	Dendritic Cell Vaccine	Phase 1	Active, not recruiting
NCT02090101	Metastatic Colorectal Cancer	32 patients	LV5FU2 Bevacizumab+Anakinra	Phase 2	Completed
NCT04796220	Breast Cancer	48 patients	Focused Ultrasound and Gemcitabine	Phase 1	Not yet recruiting
NCT02603003	Non-small Cell Lung Cancer	218 patients	JinFuKang, Cisplatin and Pemetrexed	Phase 1	Completed
NCT04170556	Hepatocellular Carcinoma	78 patients	Regorafenib and Nivolumab	Phase 1/2	Recruiting
NCT03245489	Head and Neck Squamous Cell Carcinoma	20 patients	Pembrolizumab in Combination With Anti-platelet Therapy	Phase 1	Recruiting
NCT04361409	Head and Neck Squamous Cell Carcinoma	10 patients	Rituximab Plus Chemotherapy	Phase 1	Completed

NCT02830594	Esophagus, Stomach, or Gastroesophageal Junction Cancer	14 patients	Pembrolizumab and Palliative Radiation Therapy	Phase 2	Active, not recruiting
NCT03801304	Non-small Cell Lung Cancer	80 patients	Vinorelbine With Metronomic Administration in Combination With Atezolizumab	Phase 2	Active, not recruiting
NCT03854799	Locally Advanced Rectal Cancer	101 patients	Avelumab and Capecitabine	Phase 2	Recruiting
NCT02157051	HER2-Negative Stage III-IV Breast Cancer	40 patients	CD105/Yb-1/SOX2/CDH3/MDM2-polyepitope Plasmid DNA Vaccine	Phase 1	Recruiting
NCT04028245	Localized Clear Cell Renal Cell Carcinoma	14 patients	Combination Spartalizumab and Canakinumab	Phase 1	Recruiting
NCT04919369	Recurrent or Metastatic Non-Small Cell Lung Cancer	18 patients	All-Trans Retinoic Acid (ATRA) and Atezolizumab	Phase 1	Not yet recruiting
NCT03964337	High-risk prostate cancer	33 patients	Prostatectomy vs. Cabozantinib Followed by Prostatectomy	Phase 2	Recruiting
NCT01413022	Locally Advanced Pancreatic Adenocarcinoma	44 patients	FOLFIRINOX Plus PF-04136309	Phase 1	Completed
NCT03269526	Pancreatic Cancer	22 patients	EGFR BATs	Phase 1/2	Recruiting
NCT01953900	Osteosarcoma Neuroblastoma	26 patients	iC9-GD2-CAR-VZV-CTLs	Phase 1	Active, not recruiting
NCT02275039	Ovarian Epithelial Cancer	12 patients	p53MVA Vaccine and Gemcitabine Hydrochloride	Phase 1	Completed
NCT03203005	Hepatocellular Carcinoma	22 patients	IMA970A Plus CV8102	Phase 1/2	Completed
NCT02479230	Metastatic Breast Cancer	18 patients	Type I-Polarized Autologous Dendritic Cell Vaccine With Tumor Blood Vessel Antigen-Derived Peptides	Phase 1	Completed
NCT04068649	Metastatic Malignant Neoplasm	1500 patients	Single-Fraction SBRT Versus Standard Palliative Radiation Therapy	Phase 2	Recruiting
NCT03410030	Pancreatic Cancer	27 patients	Ascorbic Acid (AA) + Nanoparticle Paclitaxel Protein Bound + Cisplatin + Gemcitabine	Phase 1/2	Active, not recruiting
NCT04011033	Advanced HCC	144 patients	Adoptive Transfer of iNKT Cells Combined With TACE	Phase 2/3	Recruiting
NCT01808820	Malignant Glioma and Glioblastoma	20 patients	Dendritic Cell (DC) Vaccine	Phase 1	Active, not recruiting
NCT02961257	Prostate Cancer Metastatic	170 patients	cabazitaxel, Prednisone, Granulocyte colony-stimulating factor (G-CSF)	Phase 3	Recruiting
NCT03618641	Melanoma	34 patients	CMP-001 in Combo With Nivolumab	Phase 2	Active, not recruiting
NCT04375956	Ovarian, Primary Peritoneal, or Fallopian Tube Cancer	100 patients	Pembrolizumab	Phase 2	Not yet recruiting
NCT04599140	Colorectal Cancer	53 patients	SX-682 and Nivolumab	Phase 1/2	Recruiting
NCT04848519	Recurrent or Metastatic Urothelial Cancer	25 patients	Propranolol Hydrochloride and Pembrolizumab	Phase 2	Recruiting
NCT04323202	Advanced Basal Cell Carcinoma	15 patients	Neoadjuvant-Adjuvant Pembrolizumab	Phase 1	Recruiting
NCT03311308	Advanced Melanoma	30 patients	Pembrolizumab and Metformin Versus Pembrolizumab	Phase 1	Recruiting
NCT02917772	Metastatic or Advanced Renal Cell Carcinoma	200 patients	Nivolumab/Ipilimumab	Phase 2	Active, not recruiting
NCT04616248	Breast Cancer	36 patients	Radio-immunotherapy (CDX-301, Radiotherapy, CDX-1140 and Poly-ICLC)	Phase 1	Recruiting
NCT03201458	Metastatic Bile Duct Cancer	76 patients	Atezolizumab With or Without Cobimetinib	Phase 2	Active, not recruiting
NCT04986852	Metastatic Triple-Negative Breast Cancer	36 patients	Olinvacimab With Pembrolizumab	Phase 2	Not yet recruiting

NCT04722575	Melanoma	88 patients	Combination or Sequence of Vemurafenib, Cobimetinib, and Atezolizumab	Phase 2	Recruiting
NCT01684397	Kidney Cancer	51 patients	Pazopanib Hydrochloride and Bevacizumab	Phase 1/2	Recruiting
NCT04612530	Metastatic Pancreatic Cancer	18 patients	Irreversible Electroporation (IRE) + Nivolumab + CpG	Phase 1	Recruiting
NCT02718443	Glioblastoma	14 patients	VXM01	Phase 1	Completed
NCT04079712	Poorly Differentiated Neuroendocrine Tumors	30 patients	Combination of XL184 (Cabozantinib), Nivolumab, and Ipilimumab	Phase 2	Recruiting
NCT03113487	Ovarian, Primary Peritoneal, or Fallopian Tube Cancer	28 patients	P53MVA and Pembrolizumab	Phase 2	Recruiting
NCT04274023	Advanced Clear Cell Sarcoma	16 patients	TSR-042	Phase 2	Active, not recruiting
NCT04015700	Unmethylated Glioblastoma	12 patients	Neoantigen-based Personalized DNA Vaccine	Phase 1	Recruiting
NCT03200847	Advanced Melanoma	26 patients	Pembrolizumab and All-Trans Retinoic Acid Combination	Phase 1/2	Active, not recruiting
NCT04539366	Osteosarcoma and Neuroblastoma	67 patients	GD2-Targeted Modified T-cells (GD2CART)	Phase 1	Not yet recruiting
NCT04835402	Pancreas Cancer	16 patients	Electroporation Potentiated Immunotherapy	Phase 2	Recruiting
NCT02957968	Breast Cancer	32 patients	Neoadjuvant Pembrolizumab + Decitabine	Phase 2	Recruiting
NCT03447678	Non Small Cell Lung Cancer	65 patients	Pembrolizumab	Phase 2	Active, not recruiting
NCT02406183	Melanoma	13 patients	SBRT With Concurrent Ipilimumab	Phase 1	Completed
NCT01720836	Non-small Cell Lung Cancer	30 patients	Vaccine + PolyICLC	Phase 1/2	Recruiting
NCT02134925	Advanced Colon Polyps	110 patients	MUC1 Peptide-Poly-ICLC Vaccine	Phase 2	Active, not recruiting
NCT03961698	Triple-Negative Breast Cancer or Renal Cell Carcinoma	90 patients	IPI-549 Combined With Front-line	Phase 2	Recruiting
NCT03680521	Clear Cell Renal Cell Carcinoma	25 patients	Neoadjuvant Sitravatinib in Combination With Nivolumab	Phase 2	Active, not recruiting
NCT02620865	Pancreatic Cancer	2 patients	Bispecific Antibody Armed Activated T-cells With Aldesleukin and Sargramostim	Phase 1/2	Active, not recruiting
NCT03177187	Metastatic Castration Resistant Prostate Cancer	86 patients	AZD5069 and Enzalutamide	Phase 1/2	Recruiting
NCT04909034	Non-small Cell Lung Cancer	30 patients	MS-20 In Combination With Pembrolizumab	Phase 2	Not yet recruiting
NCT03415854	Pancreatic Adenocarcinoma	14 patients	Paclitaxel Protein Bound Plus Cisplatin Plus Gemcitabine and Paricalcitol	Phase 2	Active, not recruiting
NCT03829111	Advanced Kidney Cancer	30 patients	CBM588, Nivolumab, and Ipilimumab	Phase 1	Recruiting
NCT02856815	Hepatocellular Carcinoma	78 patients	Immune cell-LC	Phase 2	Active, not recruiting
NCT02718430	Colorectal Cancer With Liver Metastasis	6 patients	VXM01	Phase 1	Completed
NCT03089606	Melanoma	27 patients	Pembrolizumab and PET scan	Phase 2	Active, not recruiting
NCT01935921	Head and Neck Cancer	19 patients	Ipilimumab, Cetuximab, and Intensity-Modulated Radiation Therapy	Phase 1	Active, not recruiting
NCT04931017	Lung Cancer	50 patients	Extended Release Metformin Hydrochloride	Phase 2	Not yet recruiting
NCT04856176	Non-small Cell Lung Cancer	83 patients	Sargramostim Plus Pembrolizumab With or Without Pemetrexed	Phase 2	Not yet recruiting
